# Coptidis rhizoma and its main bioactive components: recent advances in chemical investigation, quality evaluation and pharmacological activity

**DOI:** 10.1186/s13020-018-0171-3

**Published:** 2018-03-07

**Authors:** Fan-Cheng Meng, Zheng-Feng Wu, Zhi-Qi Yin, Li-Gen Lin, Ruibing Wang, Qing-Wen Zhang

**Affiliations:** 1 State Key Laboratory of Quality Research in Chinese Medicine, Institute of Chinese Medical Sciences, University of Macau, Macao SAR, People’s Republic of China; 2 Department of Traditional Chinese Medicines Pharmaceuticals, China Pharmaceutical University, Nanjing, 210009 People’s Republic of China

**Keywords:** Coptidis rhizoma, *Coptis* genus, Phytochemistry, Quality evaluation, Pharmacological effects

## Abstract

**Background:**

Coptidis rhizoma (CR) is the dried rhizome of *Coptis chinensis* Franch., *C. deltoidea* C. Y. Cheng et Hsiao or *C. teeta* Wall. (Ranunculaceae) and is commonly used in Traditional Chinese Medicine for the treatment of various diseases including bacillary dysentery, typhoid, tuberculosis, epidemic cerebrospinal meningitis, empyrosis, pertussis, and other illnesses.

**Methods:**

A literature survey was conducted via SciFinder, ScieneDirect, PubMed, Springer, and Wiley databases. A total of 139 selected references were classified on the basis of their research scopes, including chemical investigation, quality evaluation and pharmacological studies.

**Results:**

Many types of secondary metabolites including alkaloids, lignans, phenylpropanoids, flavonoids, phenolic compounds, saccharides, and steroids have been isolated from CR. Among them, protoberberine-type alkaloids, such as berberine, palmatine, coptisine, epiberberine, jatrorrhizine, columamine, are the main components of CR. Quantitative determination of these alkaloids is a very important aspect in the quality evaluation of CR. In recent years, with the advances in isolation and detection technologies, many new instruments and methods have been developed for the quantitative and qualitative analysis of the main alkaloids from CR. The quality control of CR has provided safety for pharmacological applications. These quality evaluation methods are also frequently employed to screen the active components from CR. Various investigations have shown that CR and its main alkaloids exhibited many powerful pharmacological effects including anti-inflammatory, anti-cancer, anti-diabetic, neuroprotective, cardioprotective, hypoglycemic, anti-Alzheimer and hepatoprotective activities.

**Conclusion:**

This review summarizes the recent phytochemical investigations, quality evaluation methods, the biological studies focusing on CR as well as its main alkaloids.

## Background

Coptidis rhizoma (CR) is the dried rhizome of *Coptis chinensis* Franch., *C. deltoidea* C. Y. Cheng et Hsiao or *C. teeta* Wall. (Ranunculaceae). The rhizomes of *C. japonica* Makino and *C. japonica var. dissecta* are also used as CR in Japan. CR has a long history of usage for clearing heat, eliminating dampness, purging fire and detoxification in Traditional Chinese Medicine (TCM). CR, also called goldthread, was frequently used for the treatment of bacillary dysentery, typhoid, tuberculosis, epidemic cerebrospinal meningitis, empyrosis, pertussis, and other diseases.

Chemical investigations have led to the discovery of multiple secondary metabolites including alkaloids, lignans, phenylpropanoids, flavonoids, phenolic acids, saccharides and steroids in CR. Protoberberine-type alkaloids, such as berberine, palmatine, coptisine, epiberberine, jatrorrhizine and columamine, are the main bioactive components of CR.

Achieving a high degree of quality control is very important to ensure the safety and efficacy of TCM. Recent technological advances have made great progress in the quantitative and qualitative analysis of the main alkaloids extracted from CR. The applications of High Performance Liquid Chromatography (HPLC) or Ultra Performance Liquid Chromatography (UPLC) combined with Mass Spectrometry (MS) or MS^n^ [1, 2] and quantitative ^1^H-NMR [3] are the most attractive strategies of ensuring quality control. Besides, these quality evaluation methods were also frequently employed to screen the pharmaceutically active components derived from CR [4, 5].

Various pharmacological investigations have indicated that CR and its main alkaloids exhibit many biological activities including anti-inflammatory, anti-cancer, hypoglycemic, anti-diabetic, neuroprotective and cardioprotective effects [6–12]. In this paper, we have summarized the phytochemical investigations, quality evaluation methods, and biological studies of CR as well as its main alkaloids that have been developed and conducted in recent years.

## Phytochemical investigation

Previous phytochemical investigations on CR have led to the isolation and identification of many types of natural products including alkaloids, lignans, phenylpropanoids, flavonoids, phenolic compounds, saccharides and steroids. Up to this point, phytochemical investigations have focused on *C. chinensis*, *C. Japonica* var. *dissecta*, *C. teeta* and the main components of the plants from *Coptis* genus were found to be similar while the minor constituents differed.

Classic column chromatography utilizing silica gel as the stationary phase was widely used for the isolation of constituents from CR. Column chromatography over Sephadex LH-20, polyamide and octadecylsilane (ODS) was also used as well as preparative HPLC equipped with a reversed phase column or a chiral column. In general, it has often been difficult to isolate alkaloids from CR. However, through the application of suitable solvent systems in high-speed countercurrent chromatography, such as *n*-hexane–ethyl-acetate–methanol–water (2:5:2:5) [13] and chloroform–methanol–water (4:3:3, v/v) with HCl (60 mM) and triethylamine (5 mM) added into the upper aqueous phase and the lower organic phase [14], the highly pure main alkaloids could be readily separated and obtained in considerable yields. Spectroscopic analyses were employed in structural determination.

### Alkaloids

**Fig. 1:**
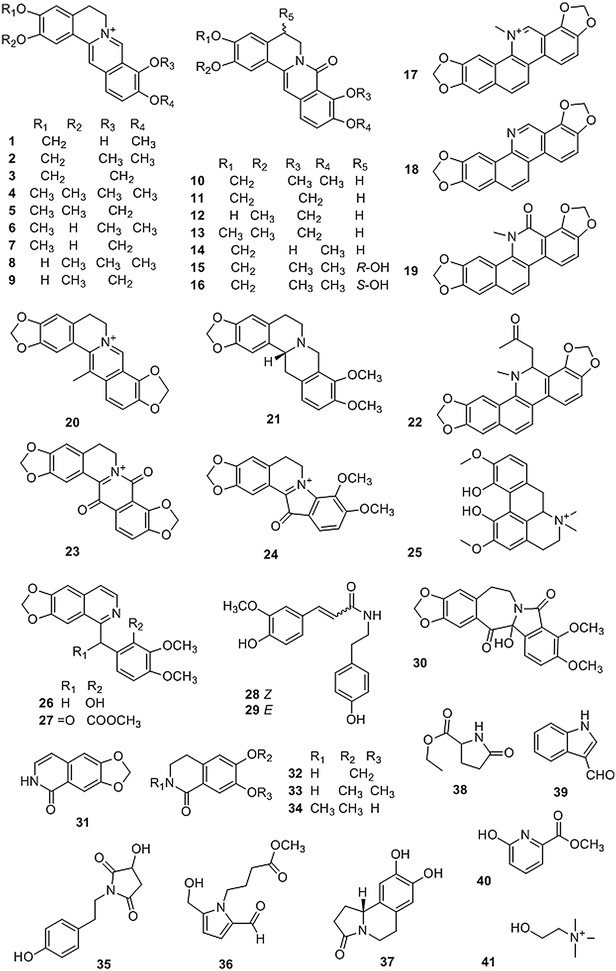
Structures of alkaloids isolated from *Coptis* genus

Alkaloids are the main components of CR (Fig. 1 and Table 1), of which protoberberine alkaloids are most common in plants from the *Coptis* genus. Most of them are isoquinolines and quaternary iminium type alkaloids. So far, the protoberberine alkaloids reported from CR include berberine-type (**1**–**9**) [15], oxyberberine-type (**10**–**16**, **23**–**24, 30**), methyl berberine type (**20**), and protoberberine-type (**21**) alkaloids. Benzophenanthridines (**17**–**19, 22**), aporphine (**25**), benzylisoquinolines (**26**–**27**), phenethylamines (**28**–**29**, **35**) and isoquinolines (**31**–**34**) were also the main alkaloid components of various species of *Coptis* genus. Additionally, some other nitrogen-containing molecules were also isolated from *C. chinensis* [16–29].

**Table 1:** Alkaloids isolated from *Coptis* genus

No.	Compounds	Source	Rfs.
**1**	Berberrubine	*C. chinensis*	[15, 16]
**2**	Berberine	*C. chinensis*	[16–22]
**3**	Coptisine	*C. chinensis*	[16–18, 21]
**4**	Palmatine	*C. chinensis*	[15, 17, 18, 21]
**5**	Epiberberine	*C. chinensis*	[15–18, 21]
**6**	Columbamine	*C. chinensis*	[15, 17, 18]
**7**	Tetradehydroscoulerine	*C. chinensis*	[17]
**8**	Jatrorrhizine	*C. chinensis*	[15, 17, 18]
**9**	Groenlandicine	*C. chinensis*	[15–18]
**10**	8-Oxyberberine	*C. chinensis*	[15, 19–24]
**11**	8-oxo-Coptisine	*C. chinensis*	[15, 18, 19, 21–24]
**12**	3-Hydroxy-2-methoxy-9,10-methylenedioxy-8-oxo-Protoberberine	*C. chinensis*	[24]
**13**	8-oxo-Epiberberine	*C. chinensis*	[22, 23]
**14**	8-Oxyberberrubine	*C. chinensis*	[22]
**15**	(−)-5-Hydroxyl-8-oxyberberine	*C. chinensis*	[21]
**16**	(+)-5-Hydroxyl-8-oxyberberine	*C. chinensis*	[21]
**17**	Sanguinarine	*C. Japonica* var. *dissecta*	[25]
**18**	Norsanguinarine	*C. Japonica* var. *dissecta*	[25]
**19**	Oxysanguinarine	*C. Japonica* var. *dissecta*	[25]
**20**	Worenine	*C. chinensis*	[26]
**21**	Tetrahydroberberine	*C. chinensis*	[21]
**22**	6-Acetonyl-5,6-dihydrosanguinarine	*C. Japonica* var. *dissecta*	[25]
**23**	8,13-Dioxocoptisine hydroxide	*C. chinensis*	[22]
**24**	Coptisonine	*C. chinensis*	[22]
**25**	Magnoflorine	*C. chinensis*	[15, 17, 18]
**26**	Berbithine	*C. chinensis*	[21, 22]
**27**	6-([1,3]Dioxolo[4,5-g]isoquinoline-5-carbonyl)-2,3-dimethoxy benzoic acid methyl ester	*C. chinensis*	[21, 23]
**28**	*N*-*cis*-Ferulyltyramine	*C. chinensis*	[27]
**29**	*N*-*tran*-Feruloyltyramine	*C. chinensis*	[20, 28]
**30**	Chilenine	*C. chinensis*	[22, 28]
**31**	1,3-Dioxolo[4,5-g]isoquinolin-5(6*H*)-one	*C. chinensis*	[23]
**32**	Noroxyhydrastinine	*C. chinensis*	[19, 22, 23]
**33**	Corydaldine	*C. chinensis*	[19, 22, 23]
**34**	Thalifoline	*C. chinensis*	[27]
**35**	3-Hydroxy-1-(4-hydroxyphenethyl) pyrrolidine-2,5-dione	*C. chinensis*	[29]
**36**	4′-[Formyl-5-(hydroxymethyl)-1H-pyrrol-1-yl] butanoate	*C. chinensis*	[28]
**37**	8,9-Dihydroxy-1,5,6,10b-tetrahydro-2*H*-pyrrolo[2,1-α]-isoquinolin-5-one	*C. chinensis*	[26]
**38**	Ehyl-2-pyrrolidinone-5(*S*)-carboxylate	*C. chinensis*	[27]
**39**	Methyl-5-hydroxy-2-pyridinecarboxylate	*C. chinensis*	[27]
**40**	1*H*-Indole-3-carboxaldehyde	*C. chinensis*	[27]
**41**	Choline	*C. chinensis*	[15]

### Lignans

**Fig. 2:**
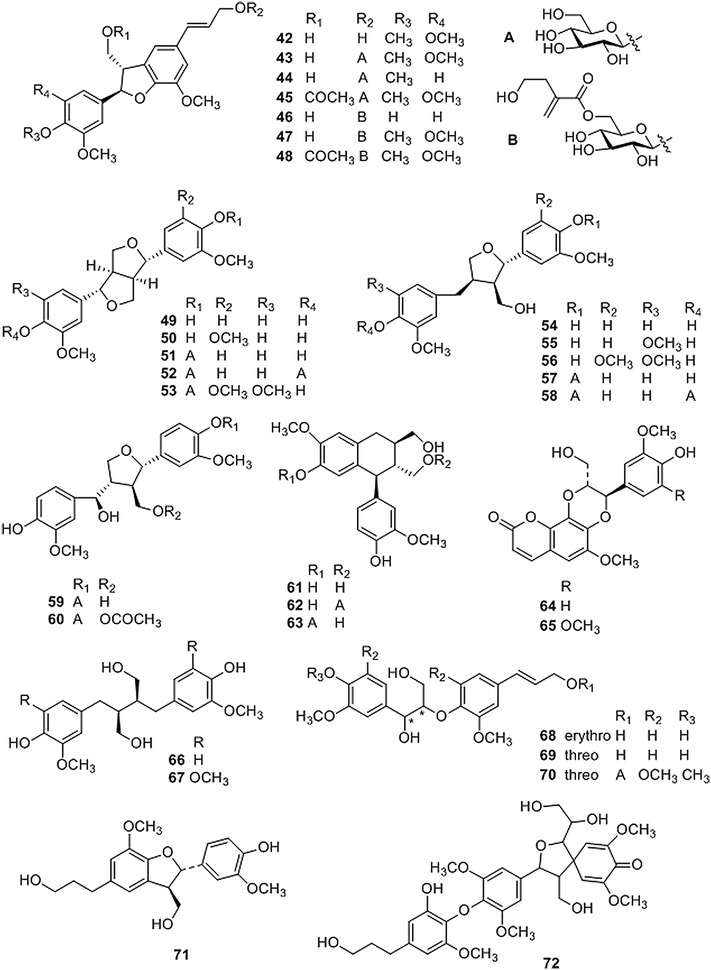
Structures of lignans isolated from *Coptis* genus

Lignans (Fig. 2 and Table 2) are also abundant in CR and have a wide variety of structures which can be classified into several skeletal types, such as benzofurans (**42**–**48**), furofurans (**49**–**53**), tetrahydrofurans (**54**–**60**), arylnaphthanlenes (**61**–**63**) and others (**64**–**72**) [18, 20, 26, 27, 30–38].

**Table 2:** Lignans isolated from *Coptis* genus

No.	Compounds	Source	Rfs.
**42**	Woorenogenin	*C. chinensis*; *C. japonica* var. *dissecta*	[30, 31]
**43**	Woorenoside I	*C. japonica* var. *dissecta*; *C. teeta*	[31, 32]
**44**	Longifolroside A	*C. teeta*	[32]
**45**	Woorenoside II	*C. japonica* var. *dissecta*; *C. teeta*	[31, 32]
**46**	Woorenoside V	*C. japonica* var. *dissecta*	[31]
**47**	Woorenoside III	*C. japonica* var. *dissecta*	[31]
**48**	Woorenoside IV	*C. japonica* var. *dissecta*	[31]
**49**	(+)-Pinoresinol	*C. chinensis*; *C. japonica* var. *dissecta*	[31, 33]
**50**	(+)-Medioresinol	*C. chinensis*	[33]
**51**	(+)-Pinoresinol glucoside	*C. japonica* var. *dissecta*	[31]
**52**	(+)-Pinoresinol-4,4′-*O*-*β*-d-diglucopyranoside	*C. chinensis*; *C. japonica* var. *dissecta*	[18, 34]
**53**	(+)-Syringaresinol glucoside	*C. japonica* var. *dissecta*; *C. teeta*	[32]
**54**	(+)-Lariciresinol	*C. chinensis*; *C. teeta*	[20, 32, 33, 35]
**55**	(±)-5,5′-Dimethoxylariciresinol	*C. chinensis*	[26]
**56**	(+)-5′-Methoxylariciresinol	*C. chinensis*	[33]
**57**	(+)-Lariciresinol glucoside	*C. chinensis*; *C. japonica* var. *dissecta*	[18, 30, 31]
**58**	7*S*,8*R*,8′*R*-(+)-Lariciresinol-4,4′-*O*-*β*-d-diglucopyranoside	*C. chinensis*; *C. japonica* var. *dissecta*	[18, 34]
**59**	Lanicepside A	*C. chinensis*	[30]
**60**	9-Acetyl lanicepside B	*C. chinensis*	[30]
**61**	(+)-Isolariciresinol	*C. chinensis*; *C. japonica* var. *dissecta*	[30, 31, 33]
**62**	Isolarisiresinol-9-*O*-*β*-d-glucopyranoside	*C. chinensis*	[18]
**63**	Woorenoside XI	*C. japonica* var. *dissecta*	[34]
**64**	Cleomiscosin A	*C. japonica* var. *dissecta*	[36]
**65**	Aquillochin	*C. japonica* var. *dissecta*	[36]
**66**	2,3-bis[(4-Hydroxy-3,5-dimethoxyphenyl)-methyl]-1,4-butanediol	*C. chinensis*	[37]
**67**	Secoisolariciresinol	*C. chinensis*	[27]
**68**	*erythro*-Guaiacylglycerol-8-*O*-4′-(coniferyl alcohol) ether	*C. chinensis*	[33]
**69**	*threo*-Guaiacylglycerol-8-*O*-4′-(coniferyl alcohol) ether	*C. chinensis*	[33]
**70**	Woorenoside X	*C. japonica* var. *dissecta*	[34]
**71**	Dihydrodehydrodiconiferyl alcohol	*C. chinensis*	[37]
**72**	Woorenol	*C. japonica* var. *dissecta*	[38]

### Simple phenylpropanoids

**Fig. 3:**
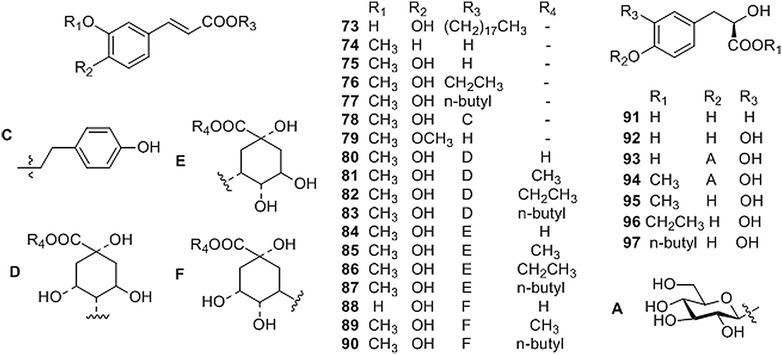
Structures of phenylpropanoids isolated from *Coptis* genus

Simple phenylpropanoids with a mother-nucleus of phenylpropionic acid have been isolated from CR (Fig. 3 and Table 3). The esterified derivatives of these phenylpropionic acids bearing methyl, ethyl, *n*-butyl, quinic acid, etc. moieties were also isolated from the plants of CR [18–20, 22, 26–28, 31–35, 37, 39].

**Table 3:** Phenylpropanoids isolated from *Coptis* genus

No.	Compounds	Source	Rfs.
**73**	*Z*-Octadecyl caffeate	*C. chinensis*; *C. teeta*	[22, 32, 33]
**74**	*E*-3-Methoxycinnamic acid	*C. chinensis*	[19]
**75**	Ferulic acid	*C. chinensis*; *C. teeta*	[18, 32, 33]
**76**	Ethyl ferulate	*C. chinensis*; *C. japonica* var. *dissecta*	[31]
**77**	*n*-Butyl ferulate	*C. chinensis*	[19]
**78**	*p*-Hydroxyphenethyl trans-ferulate	*C. japonica*	[35]
**79**	*E*-3,4-Dimethoxycinnamic acid	*C. chinensis*	[19]
**80**	4-*O*-Feruloylquinic acid	*C. chinensis*	[18, 39]
**81**	Methyl 4-*O*-feruloylquicinate	*C. japonica* var. *dissecta*	[34]
**82**	Ethyl 4-*O*-feruloylquicinate	*C. chinensis*	[37]
**83**	4-*O*-Feruloylquinic acid butyl ester	*C. chinensis*	[19]
**84**	5-*O*-Feruloylquinic acid	*C. chinensis*	[18, 39]
**85**	Methyl 5-*O*-feruloylquicinate	*C. chinensis*	[26]
**86**	Ethyl 5-*O*-feruloylquicinate	*C. chinensis*	[26]
**87**	5-*O*-Feruloylquinic acid butyl ester	*C. chinensis*	[19]
**88**	Chlorogenic acid	*C. chinensis*	[33]
**89**	Methyl 3-*O*-feruloylquicinate	*C. chinensis*	[27, 28, 37]
**90**	*n*-Butyl 3-*O*-feruloylquicinate	*C. chinensis*	[27, 28]
**91**	3-(4′-Hydroxyphenyl)-(2*R*)-lactic acid	*C. chinensis*	[18]
**92**	3-(3′,4′-Hydroxyphenyl)-(2*R*)-lactic acid	*C. chinensis*	[18, 39]
**93**	3-(3′,4′-Dihydroxyphenyl)-(2*R*)-lactic acid-4′-*O*-*β*-d-glucopyranoside	*C. chinensis*	[18, 39]
**94**	Methyl-3-(4′-O-β-d-glucopyranosyl-3′,4′-dihydroxyphenyl)-lactate	*C. japonica* var. *dissecta*	[34]
**95**	Methyl-3,4-dihydroxyphenyl lactate	*C. chinensis* *C. teeta*	[18, 20, 28, 32]
**96**	Ethyl-3,4-dihydroxyphenyl lactate	*C. chinensis*	[28]
**97**	*n*-Butyl-3,4-dihydroxyphenyl lactate	*C. chinensis*	[19]

### Flavonoids

**Fig. 4:**
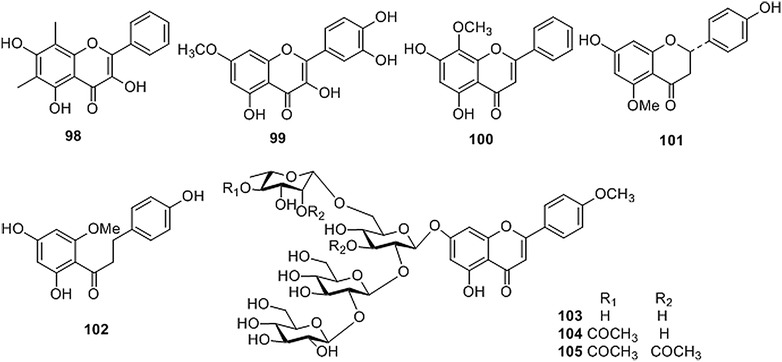
Structures of flavonoids isolated from *Coptis* genus

Plants of the *Coptis* genus also contain flavonoids [33, 34, 36, 40]. Up to now, 8 flavonoids (Fig. 4 and Table 4) have been isolated from CR. Meng [32] reported a 6,8-dimethyl substituted flavonol (**98**) that was isolated from the rhizome of *C. teeta*. Chen et al. [33] purified a flavonol (**99**) and a flavone (**100**) from the rhizome of *C. chinensis*. In earlier chemical studies, a flavanone (**101**) and a dihydrochalcone (**102**) were extracted from the seeds of *C. japonica*, and three flavonoid glycosides (**103**–**105**) were isolated from the leaves of *C. japonica* and rhizomes of *C. japonica* var. *dissecta* [34, 36, 40].

**Table 4:** Flavonoids isolated from *Coptis* genus

No.	Compounds	Source	Rf.
**98**	6,8-Dimethyl-3,5,7-trihydroxyflavone	*C. teeta*	[32]
**99**	Rhamnetin	*C. chinensis*	[33]
**100**	Wogonin	*C. chinensis*	[33]
**101**	7,4′-Dihydroxy-5-methoxyflavanone	*C. japonica* var. *dissecta*	[36]
**102**	2′,4,4′-Trihydroxy-6′-methoxy-dihydrochalcone	*C. japonica* var. *dissecta*	[36]
**103**	Coptiside II	*C. japonica*	[40]
**104**	Woorenoside XII	*C. japonica* var. *dissecta*	[34]
**105**	Coptiside I	*C. japonica*	[40]

### Others

**Fig. 5:**
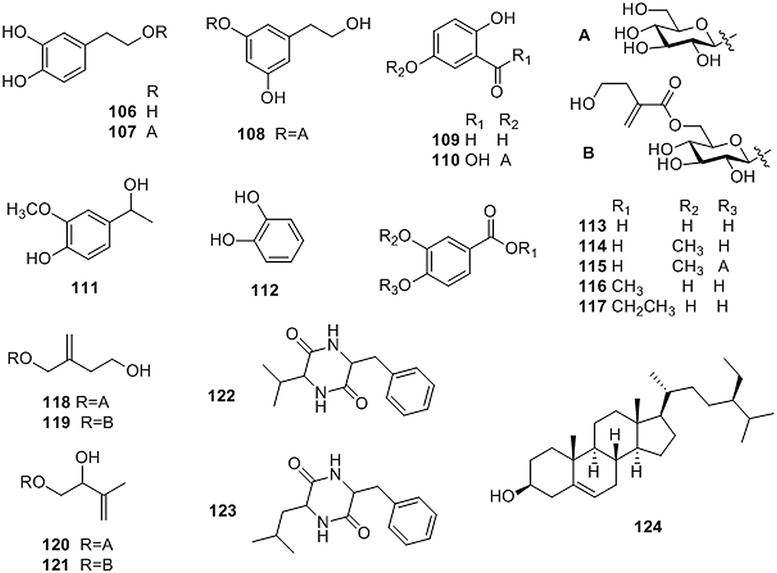
Structures of other compounds isolated from *Coptis* genus

Phenethyl alcohol and its glycosides (**106**–**108**), phenols and organic acids (**109**–**117**), hemiterpenoids (**118**–**121**), dipeptides (**122**–**123**), *β*-sitosterol (**124**) and polysaccharides were also isolated from CR [18–20, 22, 26–28, 32, 34, 37, 39]. The structures of these compounds are shown in Fig. 5, while their corresponding references and sources are summarized in Table 5.

**Table 5:** Other compounds isolated from *Coptis* genus

No.	Compounds	Source	Rfs.
**106**	3,4-Dihydroxyphenylethyl alcohol	*C. chinensis; C. teeta*	[26, 32]
**107**	3′,4′-Dihydroxyphenethyl alcohol 1-*O*-*β*-D-glucopyranoside		[39]
**108**	3,5-Dihydroxyphenethyl alcohol-3-*O*-*β*-d-glucopyranoside	*C. teeta*	[32]
**109**	Protocatechuic aldehyde	*C. chinensis*	[41]
**110**	Gentisic acid-5-*O*-*β*-d-glucopyranoside		[39]
**111**	Apocynol	*C. chinensis*	[26, 41]
**112**	1,2-Dihydroxy-benzene	*C. chinensis*	[37]
**113**	Protocatechuic acid	*C. teeta*	[32]
**114**	Vanillic acid	*C. chinensis*	[18, 20]
**115**	Vanillic acid-4-*O*-*β*-d-glucopyranoside	*C. chinensis*	[18]
**116**	Protocatechuic acid methyl ester	*C. chinensis*	[19]
**117**	Protocatechuic acid ethyl ester	*C. chinensis*	[20]
**118**	Woorenoside VI	*C. japonica var. dissecta*	[34]
**119**	Woorenoside VII	*C. japonica var. dissecta*	[34]
**120**	Woorenoside VIII	*C. japonica var. dissecta*	[34]
**121**	Woorenoside IX	*C. japonica var. dissecta*	[34]
**122**	Cyclo-(Phe-Val)	*C. chinensis*	[27]
**123**	Cyclo-(Phe-Leu)	*C. chinensis*	[27]
**124**	*β*-Sitosterol	*C. chinensis*	[22]

## Quality evaluation of CR

Quality control will play a key role in the development of TCM industry. Identification of medicinal materials is the first crucial step. DNA barcoding, a technique for species identification using standardized short gene sequences, has played an important role in the authentication herbal medicines [42]. The feasibility of identifying the species sources of CR by DNA barcoding was investigated. It was supposed that ITS2 DNA barcode could be used to identify CR and its adulterants [43]. Li et al. suggested that the combination of nuclear DNA ITS and mitochondrial DNA ycf1 could be the standard barcoding for identification of CR [44].

The protoberberine-type alkaloids, such as berberine, palmatine, coptisine, epiberberine, jatrorrhizine and columbamine, are the main components of CR [45–53] and also considered as its main bioactive compounds. The quantitative determination of these alkaloids is a very important aspect in the quality evaluation of CR. In recent years, with the advances of isolation and detection technologies, many new instrumental techniques have been employed for the quantitative and qualitative analysis of CR and the screening of the active components isolated from CR.

Achieving as complete of an extraction as possible is crucial for obtaining an accurate determination of the contents of CR. So far, refluxing or ultrasonic extraction are commonly employed in the extraction of the alkaloids from CR [45–49]. Methanol, ethanol or/and water with acid (H_2_SO_4_ or HCl) have been most frequently used as extraction solvents.

Teng and Choi [45] optimized the ultrasonic-assisted extraction method by investigating three independent variables including ethanol concentration, extraction time and the extraction temperature. Optimal extraction conditions were achieved with an ethanol concentration of 59%, extraction time of 46.57 min, and a temperature of 66.22 °C.

Methods based on HPLC are commonly used and provide a powerful tool for the quality evaluation of natural medicines. Researchers are seeking to develop new methods to extend its range of applications. Quantitative determination, differentiating the species or screening the bioactive components were achieved by thoroughly extracting information from HPLC data by using different detectors, columns, mobile phases, etc. [4, 46, 47]. Electrochemical detection was employed for the simultaneous determination of four main alkaloids of CR, and the limit of detection achieved with this technique was 80 times lower than that obtained by UV detection [48]. The application of ultra-high performance liquid chromatography greatly reduced the consumption of organic solvents and the amount of injected sample required, which saved detection time and improved the efficiency [49].

### Mass spectrometry (MS) in the quality evaluation of CR

Mass spectrometry is one of the most powerful tools for the identification of natural products, including the determination of relative molecular weights and chemical formulas, structural identification, and quantification. GC–MS was used to evaluate the volatile components of CR [50], while LC–MS has been used to detect the alkaloids present in CR [1, 2, 51–53]. An in situ reactive desorption corona beam ionization MS method was developed by Hou [48] for the direct detection of quaternary alkaloids within 1 min. The structures of the compounds were identified by analysis of their retention times, quasi-molecular ion peaks and fragment ion peaks obtained by HPLC with ESI–MS/MS methods [1] and methods for the simultaneous determination of alkaloids by MS were also developed [2]. Laser microdissection in combination with liquid chromatography–mass spectrometry (LC–MS) was employed to quantify six major alkaloids in different sections, tissues and cells of CR [52]. This method could correlate the internal quality and external features of CR from different growing areas.

### Nuclear magnetic resonance (NMR) in the quality evaluation of CR

Quantitative ^1^H-NMR (qH-NMR) has been widely used for the analysis of bioactive components in complex plant extracts in recent years [54–56]. qH-NMR has shown some advantages for quantitative analysis over traditional chromatographic methods, such as simple and easy sample preparation and handling, lower reagent consumption, elimination of the need of expensive standard compounds, and shorter analysis time. Because the C13-H signals of the main alkaloids in ^1^H NMR can be easily distinguished from each other, the quantitative determination of the main alkaloids from CR could be achieved by qH-NMR and the 3 species of CR could also be differentiated by ^1^H NMR and principal component analysis [3, 56–58].

### Others methods for quality evaluation of CR

Many other instruments and approaches have been developed for the quality evaluation of CR, such as capillary electrophoresis, ultraviolet-near infrared (UV–NIR) spectroscopy [59], Fourier transform infrared (FT-IR) spectroscopy, Raman scattering spectroscopy, as well as other methods.

A stable and reliable nanospray technique was developed by Liu et al. [60] which facilitated the differential detection of CR using CE in combination with MS. Hou et al. [61] found that surfactant-coated multi-walled carbon nanotubes, as a novel pseudostationary phase, could improve the separation resolution and selectivity of the tested isoquinoline alkaloids in nonaqueous capillary electrophoresis during the quantitative evaluation of 5 main alkaloids (coptisine, berberine, epiberberine, palmatine, and jatrorrhizine) in CR.

That the peak at 1641/cm in FT-IR of raw CR shifts to lower wave number in that of processed CR associated with second derivative IR and two-dimensional correlation IR are applied for the differentiation of various processed products and different extracts of CR [62]. He et al. [63] developed a novel method using surface enhanced Raman scattering spectroscopy to identify the geographic origins of the *C. chinensis* by analysis of the main characteristics of the Raman peaks. This method did not require elaborate separation protocols or complex data preprocessing.

## Pharmacological effects

CR, a traditional Chinese herbal medicine with potent anti-inflammatory activity, was frequently used for the treatment of bacillary dysentery, typhoid, tuberculosis, epidemic cerebrospinal meningitis, empyrosis, bronchocephalitis and other diseases [64, 65]. In recent pharmacological studies, CR and its main bioactive components, alkaloids especially berberine, have been reported to exhibit various pharmacological effects, such as anti-bacterial, anti-inflammatory, anti-oxidative, anti-tumor, anti-diabetic, hypolipidemic and neuroprotective activities [20, 22, 66–75].

### Pharmacological effects of extracts of CR

CR extracts have been studied for their variety of pharmacological activities. Protoberberine-type alkaloids are the main bioactive components of CR extracts, while other unknown ingredients may also exhibit pharmacological activities. Due to its anti-inflammatory effects, pretreatment with CR extract could decrease lipopolysaccharide (LPS)-stimulated interleukin (IL)-6 secretion, inhibit LPS-mediated nuclear factor-κB (NF-κB) activation and restore LPS-induced acute liver injury, and thus attenuate liver histopathological changes in endotoxemic mice [68]. CR could also enhance immunity by activating MOLT-4 cells and Type I helper T cells, increasing the production of Type I helper T cell cytokines including IFN-*γ*, IL-1*β*, IL-2, and IL-6 as well as by activating the mitogen-activated protein kinase (MAPKs) signaling pathways [74].

The hypolipidemic effect of CR extract may be attributed to its several functions in lipid metabolism. CR extract could promote the conversion of cholesterol into bile acids by increasing CYP7A1 activity in the liver of high lipid diet-induced hyperlipidemic rats [67]. In 3T3-L1 cells, the lipid accumulation was inhibited via the downregulation of protein levels of the adipocyte markers peroxisome proliferator-activated receptor (PPAR)-*γ* and C/EBP-*α* by CR alkaloids [76]. A modulation effect of gut microbiota of CR alkaloids may also contribute to the hypolipidemic potential [72].

CR extract exerts an anti-diabetic effect through several courses of action. CR extract had a α-glucosidase inhibitory activity with a IC_50_ value of 3.528 mg/mL, and its main alkaloids, coptisine, epiberberine, jatrorrhizin and berberine were identified as α-glucosidase inhibitors by in vitro screening [71]. The protein tyrosine phosphatase 1B inhibitory activity of berberine, epiberberine, magnoflorine and coptisine with the IC_50_ values of 16.43, 24.19, 28.14, and 51.04 μM may also contribute to the anti-diabetic effects of CR [69]. Yang [22] found that glucose uptake in differentiated C2C12 cells were increased by dichloromethane and *n*-butanol sub-fractions of CR extract at concentrations of 50 μg/mL.

CR was also reported to exhibit a neuroprotective effect against oxidative stress in human neuroblastoma cells [70] and in MPP^+^ and MPTP-induced Parkinson’s disease models [77]. The methanol and aqueous extracts of CR showed significant acetylcholinesterase inhibitory activity with IC_50_ values of 0.031 µg/mL and 2.5 µg/mL, respectively [73].

Tjong et al. [75] found that 70% ethanol extract of CR could serve as an analgesic by inhibiting the release of serotonin and cholecystokinin expression in the distal colons of rats. After treatment with an aqueous extract of CR, the elevated MDA contents were reduced and superoxide dismutase (SOD) activities were inhibited in the skin and serum of rats with radiation-induced acute skin injuries [20, 66].

### Pharmacological effects of CR-containing formulae

Traditional Chinese medicines are often used in the form of formulae and the medicines in the formulae interact with each other. Many classic formulae which contained CR have been used for 1000 of years, including Huang-Lian-Jie-Du-Decoction (Coptidis rhizoma, Scutellariae Radix, Phellodendri Cortex and Gardeniae Fructus), San-Huang-Xie-Xin-Decoction (Coptidis rhizoma, Scutellariae radix and Rhei rhizoma), Ge-Gen-Qin-Lian-Tang (Puerariae Radix, Scutellariae Radix, Coptidis rhizoma and Glycyrrhizae Radix), etc. Huang-Lian-Jie-Du-Decoction, which was frequently used for the treatment of sepsis [78], could exerted significant anti-inflammatory and anti-allergic effects [79]. It could also improve gastrointestinal motility function [80]. San-Huang-Xie-Xin-Tang has been widely used to ameliorate gastrointestinal disorders [81] and showed protective effect from neurotoxicity [82, 83] and immunomodulatory effects [84]. The traditional Chinese anti-diabetic formula, Jinqi formula (Coptidis rhizoma, *Astragali rhadix* and *Lonicerae japonicae* Flos) could inhibit TG accumulation [85]. Zuojin and Fan-Zuojin formulas with reverse mixture ratios of CR and Euodiae fructus led to different interactions on the in vitro absorption of alkaloids and opposite effects [86, 87]. Ge-Gen-Qin-Lian-Tang could decrease lipid formation [88]. Combination of Mume Fructus, Schizandrae Fructus, and CR exhibited antimicrobial activity on Enterohemorrhagic *Escherichia coli* [89]. CR and Rhei rhizoma mixture showed antioxidant and anti-inflammatory effects in Rats with reflux esophagitis [90].

### Pharmacological effects of berberine

Multiple pharmacological effects of berberine have been reported in recent years including anti-inflammatory, anti-oxidant, anti-diabetic, hypolipidemia, anti-tumor, cardiovascular protective, neuroprotective, hepatoprotective, renal protective, gut protective, and other effects [6–9, 11, 91–96]. The anti-inflammatory and anti-oxidant effects of berberine play an important role in its efficacy against various diseases, such as diabetes, hyperlipidemia and cancer by regulating the key kinases and signaling pathways [10, 12, 97, 98].

#### Inflammatory effects

The expressions of tumor necrosis factor (TNF)-*α*, IL-1*β*, IL-6, IL-17 and vascular endothelial growth factor (VEGF) were significantly reduced by berberine in the sera of rats with bovine type II collagen-induced arthritis, and the expression of VEGF and CD34 and the p-ERK, p-p38 and p-JNK activation were also inhibited [6]. Berberine could improve osteoarthritis by modulating the expression of genes and proteins related to cell proliferation, differentiation and apoptosis. In rabbit articular chondrocytes, berberine induced actin cytoskeletal architecture reorganization and dedifferentiation by inhibiting PI3-kinase/Akt as well as p38 kinase activation [99]. In osteoarthritic rat cartilage, berberine promoted cell proliferation, G1/S phase transitions and the proliferation of cell nuclear antigen synthesis via up-regulation of *β*-catenin, c-Myc, and cyclin D1 expression, and the down-regulation of glycogen synthase kinase-3*β* (GSK-3*β*) and matrix metalloproteinase-7 (MMP-7) expression [100]. Berberine could also prevent glucocorticoid-induced osteoporosis by inhibiting bone resorption and improving bone formation [101].

#### Anti-diabetic effects

The anti-inflammatory and anti-oxidant activities of berberine also play an important role in the treatment of diabetes mellitus. The ameliorating insulin resistance effect of berberine was first discovered, and then berberine was found to promote the synthesis and secretion of insulin [97, 98, 102–104]. Berberine had modulation effects on multiple kinases and signaling pathways, including 5′-adenosine monophosphate-activated protein kinase (AMPK), MAPKs, the nuclear factor erythroid-2-related factor-2 (Nrf2) pathway, and the NF-κB pathway [97, 98, 103, 104]. In the livers of high-fat diet-induced diabetic Sprague–Dawley rats, berberine could directly inhibit gluconeogenesis by decreasing the expression of gluconeogenic genes, phosphoenolpyruvate carboxykinase and glucose-6-phosphatase. Hepatic steatosis, expression of fatty acid synthase, activities of Forkhead transcription factor O1, sterol regulatory element-binding protein 1c and carbohydrate responsive element-binding protein were also inhibited in the liver by berberine [7].

#### Hypolipidemia effects

Studies indicated that the lipid-lowering effect of berberine via inhibition of cholesterol absorption, promotion of bile acid synthesis and decreasing lipid peroxidation. Berberine could significantly inhibit increases in body weight and reduce blood lipid levels in human subjects and rats [8]. It was found that treatment with berberine could up-regulate LDLR mRNA and protein expression, thus inhibiting cellular lipid accumulation in Hep G2 cells. Berberine could inhibit AMPK activity, promote brown adipose tissue formation and thereby increase energy expenditure in white and brown adipose tissue [105]. In a study conducted by Zhou et al. [106], they considered that the metabolites of berberine were the active forms due to its poor absorption and rapid metabolism.

#### Anti-tumor effects

Berberine exhibited anti-tumor activities in various cancer cell lines through inducing cell cycle arrest and apoptosis [9, 91, 107–110], inhibiting angiogenesis [109], anti-inflammation, anti-invasion and anti-metastasis [10] etc.

#### Cardiovascular protective effects

The cardiovascular protective effects of berberine were reflected by its beneficial effects in myocardial ischemia reperfusion (I/R), myocardial ischemia injury and apoptosis, cardiomyocyte hypertrophy, as well as other effects [11, 12, 92, 111–113]. In addition, berberine treatment reduced I/R induced excessive autophagy via the inhibition of SIRT1, BNIP3, and Beclin-1 expression [114].

#### Neuroprotective effects

Berberine exerts a cardiovascular protective effect by regulating the synthesis and secretion of neurotransmitters in the central nervous system. Therefore, it has a potent effect on various neurological diseases including apoplexy, depression and Alzheimer’s disease [93, 115, 116].

#### Hepatoprotective effects

Berberine has an efficacy in non-alcoholic fatty liver patients. A significant reduction of hepatic fat content and better improvement in body weight, HOMA-IR, and serum lipid profiles were displayed in patients treated with berberine and lifestyle intervention [94]. This efficacy may be related to the lipid metabolism regulatory effect of berberine. Pretreatment of berberine L02 hepatic cell lines exposed to hydrogen peroxide could increase cell viability and reduce apoptosis via the upregulation of sirtuin 1 and downregulation of apoptosis-related proteins [117].

#### Renal protective effects

Berberine could increase expressions of nephrin and podocin and exert an ameliorative effect on renal damage in high-fat diet and streptozotocin induced diabetic rats [95]. Berberine could ameliorate diabetic nephropathy via the suppression of high glucose-induced TGF-*β*1 and fibronectin synthesis in mesangial cells through an inhibition of the sphingosine kinase 1/AP-1 pathway [118].

#### Gut protective effects

In the sennoside A-induced diarrhoea mice, treatment with berberine enhanced the absorption of Na^+^ and water by improving the Na^+^/H^+^ exchanger 3 and aquaporin 4 expression, and thus ameliorated the diarrhea [96]. Berberine could also reduce the sensitivity to rectal distension and defecation of inflammatory bowel disease model rats. Berberine had an anti-nociceptive effect on visceral hypersensitivity [119]. In addition, berberine markedly elevated the activities of SOD and GSH-Px and prevented MDA, NO and villi injuries in ileum [120]. Berberine ameliorated COX-2 overexpression in the small intestinal mucosa of rats during acute endotoxemia [121].

#### Other effects

Human retinal endothelial cells (HRECs) apoptosis induced by leukocytes from diabetic patients was inhibited by berberine through a decrease in the integrin beta-2 expression of leukocytes [122]. Berberine also inhibited *Microcystis aeruginosa* growth by inducing oxidative damage [123]. Berberine acted as an efflux inhibitor and improved aminoglycoside resistance of *P. aeruginosa* [124].

### Pharmacological effects of other alkaloids from CR

Besides berberine, the main protoberberine-type alkaloids, such as palmatine, jatrorrhizine, coptisine, epiberberine, columbamine also exhibited various biological activities similar with that of berberine [125–141].

#### Palmatine

Research has shown that palmatine has modulatory effects on various cytokines and exhibits various biological activities. Ning [125] found that palmatine upregulated the mRNA and protein expressions of LDLR, CYP7A1 and downregulated ASBT mRNA and protein expressions while exhibiting a lipid-lowering effect in hamsters fed with a high-fat diet. Palmatine could regulate serum mRNA expression of TNF-*α* and IL-10, and inhibit apoptosis in mice with d-galactosamine/LPS-induced fulminant hepatic failure [126]. In osteoblast cells, palmatine could inhibit receptor activator of NF-κB ligand expression and attenuate osteoclast differentiation and function [127]. Gene reporter assays indicated that palmatine significantly activated aryl hydrocarbon receptors and elevated CYP1A1 gene promoter expression in transiently transfected HepG2 cells, which was validated in a HepG2 monolayer culture. However, no similar effects were observed in HepG2 spheroids or primary cultures of human hepatocytes [128].

Palmatine could ameliorate ischemia–reperfusion-mediated acute myocardial injuries in rats by reducing oxidative stress and modulating inflammatory mediators [129]. Among the five main alkaloids isolated from CR, palmatine showed the best AChE inhibitory activities, as demonstrated by in vitro AChE inhibition assays with IC_50_ values of 36.6 μM [130]. Jia et al. reported that West Nile virus NS2B–NS3 protease activity was inhibited by palmatine in an uncompetitive manner, with an IC_50_ value of 96 μM without detectable cytotoxicity; Furthermore, palmatine also exhibits inhibitory effects on dengue virus and yellow fever virus [131].

#### Coptisine

Coptisine could inhibit the mRNA expression of inflammatory cytokines, including nitric oxide, IL-1*β*, and IL-6 in LPS-stimulated RAW 264.7 murine macrophage cells by blocking the activation of NF-κB, MAPK and PI3K/Akt in macrophages [132]. Obesity-related inflammation was attenuated by coptisine in high fat and high cholesterol induced obese Syrian golden hamsters through LPS/TLR-4-mediated signaling pathways. Treatment with coptisine could significantly ameliorate the body weight, plasma lipid levels of TC, TG, LDL-c, VLDL-c, APoB and pro-inflammatory cytokines (TNF-*α*, IL-6, LPS) of obese hamsters. The TLR-4 in visceral fat and CD14 expression in the livers of hamsters were also suppressed [133].

The neuroprotective effect of coptisine was achieved by strengthening the thioredoxin defense system against oxidative stress and inhibition of apoptosis [134]. Coptisine could reduce myocardial injuries by attenuating the infarct size and release of MDA and increasing SOD activity. In vitro, coptisine could decrease apoptosis and protect cardiomyocytes [135].

#### Jatrorrhizine

Jatrorrhizine exhibited an inhibitory effect on the proliferation and neovascularization of metastatic melanoma cells by inducing the overexpression of cell cycle-suppressive genes p21 and p27, and cell cycle arrest at the G0/G1 transition [136]. After treatment with jatrorrhizine, the body weight of high-fat diet-induced diabetic mice was reduced, glucose tolerance and insulin sensitivity were improved and the levels of serum lipid components were ameliorated to various degrees [137]. These effects were realized by inhibiting lipogenesis and increasing lipid oxidation through the downregulation of SREBP-1c and FAS mRNA expression and induction of PPAR-*α* and CPT1A mRNA expression.

Jatrorrhizine could offset delayed gastric purging and intestinal transit via the cholinergic pathway, which was not affected by pretreatment with SB204070 in postoperative ileus rats [138]. Jatrorrhizine also had a neuroprotective effect through its anti-oxidative activity in primary rat cortical neurons [139].

#### Epiberberine

Epiberberine could inhibit 3T3-L1 adipocyte differentiation and lipid accumulation by regulating differentiation-mediated phosphorylation of factors in the Raf/MEK1/2/EREK1/2 and AMPKα/Akt pathways [140].

#### Columbamine

Columbamine had an anti-proliferative effect on metastatic osteosarcoma U2OS cells with an IC_50_ value of 21.31 ± 0.38 μM and low cytotoxicity. It could induce cyclic arrest of metastatic osteosarcoma U2OS cells at the G2/M transition by inhibiting CDK6 gene expression and STAT3 phosphorylation. Columbamine could also inhibit neovascularization of metastatic osteosarcoma U2OS cells through a down-regulation of MMP 2 expression and reduction of cell migration, adhesion, and invasion [141].

### Pharmacological effects of CR polysaccharide

Polysaccharides are polymeric carbohydrate molecules composed of more than ten monosaccharide units joined by glycosidic bonds. Natural polysaccharides are important sources of active substances. It was reported that the polysaccharides isolated from CR exhibited modest hypoglycemic effects. The *C. chinensis* polysaccharides (CCP) could increase glucose uptake in high-fat diet-induced diabetic C57bl/6J mice through lowering fasting plasma glucose levels, recovering impaired glucose tolerance and regulating the expression of glucose metabolism related genes [142]. The CCP could also inhibit the formation of advanced glycation end product (AGE) formation in vitro and in streptozotocin-induced diabetic mice. The bodyweight and serum insulin levels of streptozotocin-induced diabetic mice were significantly ameliorated and fasting blood glucose and glycated serum protein concentrations decreased. Meanwhile, the AGE accumulations and morphological abnormalities in the pancreas and liver were also improved [143]. It was also reported that the anti-diabetic effect of a water-soluble polysaccharide isolated from CR was achieved through its anti-oxidative effect involving the JNK pathway [144, 145]. This water-soluble polysaccharide CCPW-1 could increase glutathione peroxidases, SOD, catalase activities and decrease glutathione and MDA contents while inhibiting JNK expression in high-fat with streptozotocin diet induced diabetic mice. CCP also presented a protective effect against UV-induced oxidative damage [146].

## Safety and toxicity of CR

CR is usually relatively safe at normal dosages. Studies have indicated that the toxic constituents of CR were the alkaloids, and mainly berberine [147]. However, research has suggested that CR and its alkaloids could exhibit beneficial activities at low concentrations due to their increased plasma exposures [148] and enhanced intestinal absorption [149] by naturally occurring proteinaceous nanoparticles in CR extract [150]. Although CR was banned in Singapore because berberine has been associated with the aggravating effects of jaundice and kernicterus in neonates with glucose-6-phosphate dehydrogenase deficiency, in a study on 20 patients with chronic cytopenic haematological conditions, RC was administered for 1055 patient-days and no organ toxicity or electrolyte imbalance were observed [151]. Acute toxicity assays of mice showed that the LD_50_ values of berberine, coptisine, palmatine and epiberberine were 713.57, 852.12, 1533.68 and 1360 mg/kg, respectively. Their IC_50_ values in HepG2 cells were 48.17, 64.81, 112.80 and 120.58 mg/mL, and the values were 41.76, 56.48, 84.32 and 104.18 mg/mL in 3T3-L1 cells [152]. However, its toxic and adverse effects cannot be ignored. There are still many challenges in treating various diseases with CR or the alkaloids isolated from CR. Diarrhea was the most frequent toxicity effect of treatment at high dosages of CR treatment due to disturbance in the normal gut microbiota [153]. Liver and lung injuries were attributed to the fibrous root of CR at a dose of 3.76 g/kg [154]. Berberine could also act as human ether-a-go-go-related gene inhibitor which may lead to sudden death [155].

## Conclusion

Coptidis rhizoma is widely used as herbal medicine in TCM with various significant bioactivities. Until now, numerous phytochemical investigations have been carried out on CR and many types of secondary metabolites including alkaloids, lignans, phenylpropanoids, flavonoids, phenolic compounds, saccharides, steroids were reported. However, the multi-component composition of Chinese herbs and their multi-functional activities that may have greater effectiveness and more complex behavior than a single compound are also reflected in CR. Studies on the chemical components of CR are still needed to thoroughly elucidate its chemical composition and to provide a firm basis for quality control and pharmacological research. As technology has developed, more advanced instrumental methods were introduced for the quality evaluation of CR in recent years. In particular, the application of quantitative MS and quantitative NMR has provided more potential for achieving high degrees of quality control for herbal medicine. These methods will still need to be improved further to extend their applicability. Many studies have revealed that CR is a relatively safe medicine with multiple activities. The various pharmacological effects of CR and its active components also present researchers with a significant challenge to thoroughly understand their mechanisms of action. Research reveals that CR has promising potential: the bioactivities of CR were achieved by the synergistic action of multiple ingredients in the complex composition of CR. Many mechanisms underlying these pharmacological effects are still unknown and need to be discovered. To better understand the complex mechanisms that underlie the complex behavior of CR, novel research ideas and methods need to be introduced.

## Abbreviations

CR: Coptidis rhizoma
MS: mass spectrometry
HPLC: high performance liquid chromatography
UPLC: ultra high performance liquid chromatography
NMR: nuclear magnetic resonance
ODS: octadecylsilane
ESI: electrospray ionization mass spectrometry
FT-IR: Fourier transform infrared
UV: ultraviolet
ECD: electrochemical detection
QTOF: quadrupole-time of flight
GC: gas chromatography
CE: capillary electrophoresis
LPS: lipopolysaccharide
NF-Κb: nuclear factor-κB
MAPK: mitogen-activated protein kinase
SOD: superoxide dismutase
TNF: tumor necrosis factor
VEGF: vascular endothelial growth factor
MMP-7: matrix metalloproteinase-7
AMPK: 5′-adenosine monophosphate-activated protein kinase
P-TEFb: positive transcription elongation factor b
I/R: ischemia reperfusion
NOS: NO synthase
CCP: *C. chinensis* polysaccharides
AGE: advanced glycation end product

## References

[1] Ren L, Xue X, Liang X (2013). Characterization of protoberberine alkaloids in Coptidis rhizoma (Huanglian) by HPLC with ESI–MS/MS. J Sep Sci.

[2] Qian XC, Zhang L, Tao Y, Huang P, Li JS, Chai C, Li W, Di LQ, Cai BC (2015). Simultaneous determination of ten alkaloids of crude and wine-processed rhizoma Coptidis aqueous extracts in rat plasma by UHPLC–ESI–MS/MS and its application to a comparative pharmacokinetic study. J. Pharm Biomed..

[3] Fan G, Zhang MY, Zhou XD, Lai XR, Yue QH, Tang C, Luo WZ, Zhang Y (2012). Quality evaluation and species differentiation of rhizoma Coptidis by using proton nuclear magnetic resonance spectroscopy. Anal Chim Acta.

[4] Chen X, Wang J, Hu S, Bai X (2016). Hollow-fiber double-solvent synergistic microextraction with high-performance liquid chromatography for the determination of antitumor alkaloids in *Coptis chinensis*. J Sep Sci.

[5] Liu X, Hu S, Chen X, Bai X (2014). Hollow fiber cell fishing with high-performance liquid chromatography for rapid screening and analysis of an antitumor-active protoberberine alkaloid group from *Coptis chinensis*. J Pharm Biomed..

[6] Wang Z, Chen Z, Yang S, Wang Y, Huang Z, Gao J, Tu S, Rao Z (2014). Berberine ameliorates collagen-induced arthritis in rats associated with anti-inflammatory and anti-angiogenic effects. Inflammation..

[7] Xia X, Yan J, Shen Y, Tang K, Yin J, Zhang Y, Yang D, Liang H, Ye J, Weng J (2011). Berberine improves glucose metabolism in diabetic rats by inhibition of hepatic gluconeogenesis. PLoS ONE.

[8] Hu Y, Ehli EA, Kittelsrud J, Ronan PJ, Munger K, Downey T, Bohlen K, Callahan L, Munson V, Jahnke M, Marshall LL, Nelson K, Huizenga P, Hansen R, Soundy TJ, Davies GE (2012). Lipid-lowering effect of berberine in human subjects and rats. Phytomedicine.

[9] Zhang L, Miao XJ, Wang X, Pan HH, Li P, Ren H, Jia YR, Lu C, Wang HB, Yuan L, Zhang GL (2016). Antiproliferation of berberine is mediated by epigenetic modification of constitutive androstane receptor (CAR) metabolic pathway in hepatoma cells. Sci Rep..

[10] Liu X, Ji Q, Ye N, Sui H, Zhou L, Zhu H, Fan Z, Cai J, Li Q (2015). Berberine inhibits invasion and metastasis of colorectal cancer cells via COX-2/PGE2 mediated JAK2/STAT3 signaling pathway. PLoS ONE.

[11] Zhu QW, Li YG (2016). Berberine attenuates myocardial ischemia reperfusion injury by suppressing the activation of PI3K/AKT signaling. Exp Ther Med..

[12] Liu X, Zhang X, Ye L, Yuan H (2016). Protective mechanisms of berberine against experimental autoimmune myocarditis in a rat model. Biomed Pharmacother.

[13] Zhang S, Wang M, Wang C (2011). Preparative separation and purification of alkaloids from rhizoma Coptidis by high-speed counter-current chromatography. Sep Purif Technol.

[14] Sun C, Li J, Wang X, Duan W, Zhang T, Ito Y (2014). Preparative separation of quaternary ammonium alkaloids from Coptis chinensis Franch by pH-zone-refining counter-current chromatography. J Chromatogr A.

[15] Chen HY, Ye XL, Cui XL, He K, Jin YN, Chen Z, Li XG (2012). Cytotoxicity and antihyperglycemic effect of minor constituents from rhizoma Coptis in HepG2 cells. Fitoterapia.

[16] Kobayashi Y, Yamashita Y, Fujii N, Takaboshi K, Kawakami T, Kawamura M, Mizukami T, Nakano H (1995). Inhibitors of DNA topoisomerase I and II. isolated from the Coptis Rhizomes. Planta Med.

[17] Chen J, Wang F, Liu J, Lee FS, Wang X, Yang H (2008). Analysis of alkaloids in *Coptis chinensis* Franch by accelerated solvent extraction combined with ultra performance liquid chromatographic analysis with photodiode array and tandem mass spectrometry detections. Anal Chim Acta.

[18] Li XG, Yang LG, Chen LX, Qiu F (2012). Chemical constituents from the decoction of *Coptis chinensis* Franch. J Shenyang Pharm Univ..

[19] Ma HM, Chen G, Li W, Fan XM, Li ZF, Pei YH (2011). Isolation and identification of chemical constituents from rhizoma of *Coptis chinensis*. J Shenyang Pharm Univ..

[20] Wang Q, Li ZF, Chen G, Feng YL, Fan MM, Pei YH (2012). Chemical constituents from *Coptis Chinensis* Franch. Chin J Exp Tradit Med Form..

[21] Wang L, Zhang SY, Chen L, Huang XJ, Zhang QW, Jiang RW, Yao F, Ye WC (2014). New enantiomeric isoquinoline alkaloids from *Coptis chinensis*. Phytochem Lett.

[22] Yang TC, Chao HF, Shi LS, Chang TC, Lin HC, Chang WL (2014). Alkaloids from *Coptis chinensis* root promote glucose uptake in C2C12 myotubes. Fitoterapia.

[23] Wang W, Zhang QW, Ye WC, Wang YT (2007). Isoquinoline alkaloids from the rhizoma of *Coptis chinensis*. Chin J Nat Med.

[24] Zhao M, Xian YF, Ip SP, Fong HH, Che CT (2010). A new and weakly antispasmodic protoberberine alkaloid from rhizoma Coptidis. Phytother Res..

[25] Mizuno M, Kojima H, Tanaka T, Iinuma M (1987). Benzophenanthridine alkaloids from the seeds of *Coptis japonica var. dissecta*. J Nat Prod.

[26] Li ZF, Wang Q, Feng YL, Luo XJ, Fan MM, Yang SL (2012). Chemical constituents from *Coptis chinensis*. Chin Med Mat..

[27] Li ZF, Wang Q, Feng YL, Rao Y, Yang SL, Pei YH (2012). Chemical constituents from rhizomes of *Coptis chinensis*. Chin Tradit Herbal Drugs..

[28] Ma HM, Chen G, Pei YH (2013). Isolation and identification of chemical constituents from rhizoma of *Coptis chinensis* and their cytotoxic activities. J Shenyang Pharm Univ..

[29] Li ZF, Wang Q, Chen G, Hua HM, Yang SL, Feng YL, Pei YH (2013). A new pyrrolidine derivative from the rhizome of *Coptis chinensis*. Chem Nat Compd..

[30] Chen GC, Li XL, Chen G (2016). Isolation and identification of lignans chemical constituents from *Coptis chinensis* and their inhibitory activity to protein tyrosine phosphatase-1B. Chin Pharm..

[31] Yoshikawa K, Kinoshita H, Kan Y, Arihara S (1995). Neolignans and phenylpropanoids from the rhizomes of *Coptis japonica var. dissecta*. Chem Pharm Bull.

[32] Meng FC, Wang L, Zhang J, Yin ZQ, Zhang QW, Ye WC (2013). Non-alkaloid chemical constituents from the rhizome of *Coptis teeta*. J Chin Pharm Univ..

[33] Chen L, Wang L, Zhang QW, Zhang SY, Ye WC (2012). Non-alkaloid chemical constituents from *Coptis chinensis*. Chin Mat Med..

[34] Yoshikawa K, Kinoshita H, Arihara S (1997). Non-basic components of Coptis rhizoma. II. Four new hemiterpenoid glucosides, two new phenylpropanoid glucosides and a new lavonoid glycoside from *Coptis japonica var. dissecta*. Nat Med.

[35] Hirano H, Satoshiro M, Nishioka I, Shingu T (1997). Isolation of free radical scavenger from Coptidis rhizoma. Nat Med.

[36] Mizuno M, Kojima H, Tanaka T, Iinuma M, Kimura R, Min ZD, Murata H (1987). Phenolic constituents from seeds of *Coptis japonika var. dissecta*. Phytochemistry.

[37] Li ZF, Wang Q, Feng YL, Yan YB, Fan MM, Yang SL (2012). Chemical constituents in *Coptis chinensis* Franch. Chin New Drug J..

[38] Yoshikawa K, Kinoshita H, Arihara S (1997). Woorenol, a novel Sesquineolignan with a unique spiro skeleton from the rhizomes of *Coptis japonica var. dissecta*. J Nat Prod.

[39] Yahara S, Satoshiro M, Nishioka I, Nagasawa T, Oura H (1985). Isolation and characterization of phenolic compounds from Coptidis rhizoma. Chem Pharm Bull.

[40] Fujiwara H, Nonaka G, Yagi A, Nishioka I (1976). Studies on the components of the leaves of *Coptis japonica* makino. I. the structures of coptiside I and II. Chem Pharm Bull.

[41] Ma B, Zhu L, Zang X, Chen Y, Li D, Wang Y (2013). *Coptis chinensis* inflorescence and its main alkaloids protect against ultraviolet-B-induced oxidative damage. J Funct Foods..

[42] Chen SL, Song JY, Yao H, Shi LC, Luo K, Han JP (2009). Strategy and key technique of identification of Chinese herbal medicine using DNA barcoding. Chin J Nat Med.

[43] Sun T, Sun DY, Teng SN, Lu LH, Deng CH, Li YG (2013). Identification of rhizoma Coptidis and its adulterants based on ITS2 DNA barcode. Guizhou Agric Sci.

[44] Li B, Liu J, Min DZ, He XH, Li BC, Zhu CL, Zhang ZY, Yuan QJ, Wu D (2017). DNA barcoding evaluation in the genus *Coptis salisb*. Acta Agriculturae Universitatis Jiangxiensis..

[45] Teng H, Choi YH (2014). Optimization of ultrasonic-assisted extraction of bioactive alkaloid compounds from rhizoma Coptidis (*Coptis chinensis* Franch.) using response surface methodology. Food Chem.

[46] Lv X, Li Y, Tang C, Zhang Y, Zhang J, Fan G (2016). Integration of HPLC-based fingerprint and quantitative analyses for differentiating botanical species and geographical growing origins of rhizoma Coptidis. Pharm Biol..

[47] Dai SY, Xu B, Zhang Y, Li JY, Sun F, Shi XY, Qiao YJ (2016). Establishment and reliability evaluation of the design space for HPLC analysis of six alkaloids in *Coptis chinensis* (Huanglian) using Bayesian approach. Chin J Nat Med.

[48] Liu L, Chen Z (2012). Analysis of four alkaloids of *Coptis chinensis* in rat plasma by high performance liquid chromatography with electrochemical detection. Anal Chim Acta.

[49] Jiang X, Huang LF, Wu LB, Wang ZH, Chen SL (2012). UPLC–QTOF/MS analysis of alkaloids in traditional processed *Coptis chinensis* Franch. Evid Based Complement Altern.

[50] Gao X, Yang X, Mitrevski BS, Marriott PJ (2011). Headspace solid-phase microextraction combined with GCxGC–TOFMS for the analysis of volatile compounds of *Coptis* species rhizomes. J Sep Sci.

[51] Hou Y, Wu T, Liu Y, Wang H, Chen Y, Chen B, Sun W (2014). Direct analysis of quaternary alkaloids by in situ reactive desorption corona beam ionization MS. Analyst.

[52] Yi L, Liang ZT, Peng Y, Guo P, Wong LL, Wan XJ, Ho HM, Yi T, Zhao ZZ (2015). Histochemical evaluation of alkaloids in rhizome of *Coptis chinensis* using laser microdissection and liquid chromatography/mass spectrometry. Drug Test Anal..

[53] Liu Q, Qiu S, Yu H, Ke Y, Jin Y, Liang X (2011). Selective separation of structure-related alkaloids in rhizoma Coptidis with “click” binaphthyl stationary phase and their structural elucidation with liquid chromatography–mass spectrometry. Analyst..

[54] Pauli GF, Jaki BU, Lankin DC (2005). Quantitative ^1^H NMR: development and potential of a method for natural products analysis. J Nat Prod.

[55] Tanaka R, Inagaki R, Sugimoto N, Akiyama H, Nagatsu A (2017). Application of a quantitative ^1^H-NMR (^1^H-qNMR) method for the determination of geniposidic acid and acteoside in *Plantaginis semen*. J Nat Med.

[56] Fan G, Tao LH, Yue QH, Kuang TT, Tang C, Yang YD, Luo WZ, Zhou XD, Zhang Y (2012). Metabolic discrimination of rhizoma Coptidis from different species using ^1^H NMR spectroscopy and principal component analysis. Planta Med.

[57] Hasada K, Yoshida T, Yamazaki T, Sugimoto N, Nishimura T, Nagatsu A, Mizukami H (2011). Application of ^1^H-NMR spectroscopy to validation of berberine alkaloid reagents and to chemical evaluation of Coptidis rhizoma. J Nat Med.

[58] Ding PL, Chen LQ, Lu Y, Li YG (2012). Determination of protoberberine alkaloids in rhizoma Coptidis by ERETIC ^1^H NMR method. J Pharm Biomed..

[59] Dai X, Song H, Liu W, Yao S, Wang G (2016). On-line UV–NIR spectroscopy as a process analytical technology (PAT) tool for on-line and real-time monitoring of the extraction process of Coptis Rhizome. RSC Adv..

[60] Liu JX, Zhang YW, Yuan F, Chen HX, Zhang XX (2014). Differential detection of rhizoma Coptidis by capillary electrophoresis electrospray ionization mass spectrometry with a nanospray interface. Electrophoresis.

[61] Hou J, Li G, Wei Y, Lu H, Jiang C, Zhou X, Meng F, Cao J, Liu J (2014). Analysis of five alkaloids using surfactant-coated multi-walled carbon nanotubes as the pseudostationary phase in nonaqueous capillary electrophoresis. J Chromatogr A.

[62] Xu B, Zhang G, Xu C, Sun S (2015). Analysis of fingerprints features of infrared spectra of various processed products of rhizoma Coptidis and their different extracts. J Mol Struct.

[63] He S, Liu X, Zhang W, Xie W, Zhang H, Fu W, Liu H, Liu X, Xu Y, Yang D, Gao Y (2015). Discrimination of the *Coptis chinensis* geographic origins with surface enhanced Raman scattering spectroscopy. Chemometr Intell Lab..

[64] Chinese Materia Meidca (1999). Editional committee of Chinese Materia Medica SAoTCM.

[65] Editional committee of flora of China CAoS (2004). Flroa of China.

[66] Wang XJ, Lin S, Kang HF, Dai ZJ (2013). The effect of rhizoma Coptidis and *Coptis Chinensis* aqueous extract on radiation-induced skin injury in a rat model. BMC Complem Altern M..

[67] Cao Y, Bei W, Hu Y, Cao L, Huang L, Wang L, Luo D, Chen Y, Yao X, He W, Liu X, Guo J (2012). Hypocholesterolemia of rhizoma Coptidis alkaloids is related to the bile acid by up-regulated CYP7A1 in hyperlipidemic rats. Phytomedicine.

[68] Choi YY, Kim MH, Cho IH, Kim JH, Hong J, Lee TH, Yang WM (2013). Inhibitory effect of *Coptis chinensis* on inflammation in LPS-induced endotoxemia. J Ethnopharmacol.

[69] Choi JS, Ali MY, Jung HA, Oh SH, Choi RJ, Kim EJ (2015). Protein tyrosine phosphatase 1B inhibitory activity of alkaloids from rhizoma Coptidis and their molecular docking studies. J Ethnopharmacol.

[70] Friedemann T, Otto B, Klatschke K, Schumacher U, Tao Y, Leung AK, Efferth T, Schroder S (2014). *Coptis chinensis* Franch. exhibits neuroprotective properties against oxidative stress in human neuroblastoma cells. J Ethnopharmacol.

[71] Ge AH, Bai Y, Li J, Liu J, He J, Liu EW, Zhang P, Zhang BL, Gao XM, Chang YX (2014). An activity-integrated strategy involving ultra-high-performance liquid chromatography/quadrupole-time-of-flight mass spectrometry and fraction collector for rapid screening and characterization of the α-glucosidase inhibitors in Coptis chinensis Franch. (Huanglian). J Pharm Biomed..

[72] He K, Hu Y, Ma H, Zou Z, Xiao Y, Yang Y, Feng M, Li X, Ye X (2016). Rhizoma Coptidis alkaloids alleviate hyperlipidemia in B6 mice by modulating gut microbiota and bile acid pathways. BBA Mol Basis Dis..

[73] Kaufmann D, Kaur Dogra A, Tahrani A, Herrmann F, Wink M (2016). Extracts from traditional chinese medicinal plants inhibit acetylcholinesterase, a Known Alzheimer’s disease target. Molecules.

[74] Kim E, Ahn S, Rhee HI, Lee DC (2016). *Coptis chinensis* Franch. extract up-regulate type I helper T-cell cytokine through MAPK activation in MOLT-4 T cell. J Ethnopharmacol.

[75] Tjong Y, Ip S, Lao L, Fong HH, Sung JJ, Berman B, Che C (2011). Analgesic effect of *Coptis chinensis* rhizomes (Coptidis Rhizoma) extract on rat model of irritable bowel syndrome. J Ethnopharmacol.

[76] Choi JS, Kim JH, Ali MY, Min BS, Kim GD, Jung HA (2014). *Coptis chinensis* alkaloids exert anti-adipogenic activity on 3T3-L1 adipocytes by downregulating C/EBP-*α* and PPAR-*γ*. Fitoterapia.

[77] Friedemann T, Ying Y, Wang W, Kramer ER, Schumacher U, Fei J, Schroder S (2016). Neuroprotective effect of *Coptis chinensis* in MPP^+^ and MPTP-induced Parkinson’s disease models. Am J Chin Med.

[78] Xu DQ, Lv Y, Yang MH, Wang JS, Kong LY (2017). Deciphering the mechanism of Huang-Lian-Jie-Du-Decoction on the treatment of sepsis by formula decomposition and metabolomics: enhancement of cholinergic pathways and inhibition of HMGB-1/TLR4/NF-κB signaling. Pharmacol Res..

[79] Chen YL, Xian YF, Lai ZQ, Loo S, Lin ZX, Chan WY (2016). Anti-inflammatory and anti-allergic effects and underlying mechanisms of Huang-Lian-Jie-Du extract: Implication for atopic dermatitis treatment. J Ethnopharmacol..

[80] Kim H, Kim I, Lee MC, Kim HJ, Lee GS, Kim H, Kim BJ (2017). Effects of Hwangryunhaedok-tang on gastrointestinal motility function in mice. World J Gastroenterol.

[81] Kim BJ, Kim H, Lee GS, So I, Kim SJ (2014). Effects of San-Huang-Xie-Xin-tang, a traditional Chinese prescription for clearing away heat and toxin, on the pacemaker activities of interstitial cells of Cajal from the murine small intestine. J Ethnopharmacol.

[82] Lo YC, Shih YT, Tseng YT, Hsu HT (2012). Neuroprotective effects of San-Huang-Xie-Xin-Tang in the MPP(+)/MPTP models of Parkinson’s disease in vitro and in vivo. Evid-based Compl Alt..

[83] Shih YT, Chen IJ, Wu YC, Lo YC (2011). San-Huang-Xie-Xin-Tang protects against activated microglia- and 6-OHDA-induced toxicity in neuronal SH-SY5Y cells. Evid Based Complement Altern.

[84] Li CY, Hou YC, Lee CPD, Shia CS, Hsu IC, Fang SH (2010). Potential ex vivo immunomodulatory effects of San-Huang-Xie-Xin-Tang and its component herbs on mice and humans. J Ethnopharmacol.

[85] Qian Q, Liu X, He W, An Y, Chen Q, Wu J, Deng Y, Guo L, Zhang Y, Wang T (2012). TG accumulation inhibitory effects of Jinqi formula by AMPK signaling pathway. J Ethnopharmacol.

[86] Yang YF, Zhou QL, Yang XW (2017). Elucidation of compatibility interactions of Traditional Chinese Medicines: in vitro absorptions across caco-2 monolayer of Coptidis rhizoma and Euodiae fructus in Zuojin and Fanzuojin formulas as a case. Phytother Res..

[87] Qian P, Zhang YB, Yang YF, Xu W, Yang XW (2017). Pharmacokinetics studies of 12 alkaloids in rat plasma after oral administration of Zuojin and Fan-Zuojin Formulas. Molecules..

[88] Ho FM, Liao YH, Yang AJ, Lee CPD, Hou YC, Huang CT, Lin SR, Lee KR, Huang KC, Lin WW (2012). Anti-atherosclerotic action of Ger-Gen-Chyn-Lian-Tang and AMPK-dependent lipid lowering effect in hepatocytes. J Ethnopharmacol.

[89] Lee JH, Stein BD (2011). Antimicrobial activity of a combination of Mume Fructus, Schizandrae Fructus, and Coptidis rhizoma on enterohemorrhagic *Escherichia coli* O26, O111, and O157 and its effect on shiga toxin releases. Foodborne Pathog Dis..

[90] Kwon OJ, Kim MY, Shin SH, Lee AR, Lee JY, Seo BI, Shin MR, Roh SS, Choi HG, Kim JA, Min BS, Kim GN, Noh JS, Rhee MH, Roh SS (2016). Antioxidant and anti-inflammatory effects of Rhei rhizoma and Coptidis rhizoma mixture on reflux esophagitis in Rats. Evid Based Complement Altern..

[91] Tsang CT, Cheung YC, Lui VWY, Yip YL, Zhang GT, Lin VWT, Cheung KCP, Feng YB, Tsao SW (2013). Berberine suppresses tumorigenicity and growth of nasopharyngeal carcinoma cells by inhibiting STAT3 activation induced by tumor associated fibroblasts. BMC Cancer..

[92] Chang W, Zhang M, Li J, Meng Z, Xiao D, Wei S, Chen L, Wang C, Hatch GM (2012). Berberine attenuates ischemia-reperfusion injury via regulation of adenosine-5′-monophosphate kinase activity in both non-ischemic and ischemic areas of the rat heart. Cardiovasc Drug Ther..

[93] Sun S, Wang K, Lei H, Li L, Tu M, Zeng S, Zhou H, Jiang H (2014). Inhibition of organic cation transporter 2 and 3 may be involved in the mechanism of the antidepressant-like action of berberine. Prog Neuro Psychoph..

[94] Yan HM, Xia MF, Wang Y, Chang XX, Yao XZ, Rao SX, Zeng MS, Tu YF, Feng R, Jia WP, Liu J, Deng W, Jiang JD, Gao X (2015). Efficacy of berberine in patients with non-alcoholic fatty liver disease. PLoS ONE.

[95] Wu D, Wen W, Qi CL, Zhao RX, Lu JH, Zhong CY, Chen YY (2012). Ameliorative effect of berberine on renal damage in rats with diabetes induced by high-fat diet and streptozotocin. Phytomedicine.

[96] Zhang Y, Wang X, Sha S, Liang S, Zhao L, Liu L, Chai N, Wang H, Wu K (2012). Berberine increases the expression of NHE3 and AQP4 in sennoside A-induced diarrhoea model. Fitoterapia.

[97] Chatuphonprasert W, Nemoto N, Sakuma T, Jarukamjorn K (2012). Modulations of cytochrome P450 expression in diabetic mice by berberine. Chem Biol Interact..

[98] Lo SN, Chang YP, Tsai KC, Chang CY, Wu TS, Ueng YF (2013). Inhibition of CYP1 by berberine, palmatine, and jatrorrhizine: selectivity, kinetic characterization, and molecular modeling. Toxicol Appl Pharm..

[99] Yu SM, Cho H, Kim GH, Chung KW, Seo SY, Kim SJ (2016). Berberine induces dedifferentiation by actin cytoskeleton reorganization via phosphoinositide 3-kinase/Akt and p38 kinase pathways in rabbit articular chondrocytes. Exp Biol Med..

[100] Zhou Y, Tao H, Li Y, Deng M, He B, Xia S, Zhang C, Liu S (2016). Berberine promotes proliferation of sodium nitroprusside-stimulated rat chondrocytes and osteoarthritic rat cartilage via Wnt/beta-catenin pathway. Eur J Pharmacol.

[101] Xu D, Yang W, Zhou C, Liu Y, Xu B (2010). Preventive effects of berberine on glucocorticoid-induced osteoporosis in rats. Planta Med.

[102] Zhou JY, Zhou SW (2011). Protective effect of berberine on antioxidant enzymes and positive transcription elongation factor b expression in diabetic rat liver. Fitoterapia.

[103] Ma X, Egawa T, Kimura H, Karaike K, Masuda S, Iwanaka N, Hayashi T (2010). Berberine-induced activation of 5′-adenosine monophosphate-activated protein kinase and glucose transport in rat skeletal muscles. Metabolism..

[104] Li ZQ, Zuo DY, Qie XD, Qi H, Zhao MQ, Wu YL (2012). Berberine acutely inhibits the digestion of maltose in the intestine. J Ethnopharmacol.

[105] Zhang Z, Zhang H, Li B, Meng X, Wang J, Zhang Y, Yao S, Ma Q, Jin L, Yang J, Wang W, Ning G (2014). Berberine activates thermogenesis in white and brown adipose tissue. Nature Comm..

[106] Zhou Y, Cao S, Wang Y, Xu P, Yan J, Bin W, Qiu F, Kang N (2014). Berberine metabolites could induce low density lipoprotein receptor up-regulation to exert lipid-lowering effects in human hepatoma cells. Fitoterapia.

[107] Lo TF, Tsai WC, Chen ST (2013). MicroRNA-21-3p, a berberine-induced miRNA, directly down-regulates human methionine adenosyltransferases 2A and 2B and inhibits hepatoma cell growth. PLoS ONE.

[108] Yip NK, Ho WS (2013). Berberine induces apoptosis via the mitochondrial pathway in liver cancer cells. Oncol Rep.

[109] Zhang J, Cao H, Zhang B, Cao H, Xu X, Ruan H, Yi T, Tan L, Qu R, Song G, Wang B, Hu T (2013). Berberine potently attenuates intestinal polyps growth in ApcMin mice and familial adenomatous polyposis patients through inhibition of Wnt signalling. J Cell Mol Med.

[110] Kim JB, Yu JH, Ko E, Lee KW, Song AK, Park SY, Shin I, Han W, Noh DY (2010). The alkaloid berberine inhibits the growth of Anoikis-resistant MCF-7 and MDA-MB-231 breast cancer cell lines by inducing cell cycle arrest. Phytomed Int J Phytother Phytopharmacol..

[111] Huang Z, Han Z, Ye B, Dai Z, Shan P, Lu Z, Dai K, Wang C, Huang W (2015). Berberine alleviates cardiac ischemia/reperfusion injury by inhibiting excessive autophagy in cardiomyocytes. Eur J Pharmacol.

[112] Lv XX, Yu XH, Wang YY, Wang FQ, Li HM (2012). Wang Yp, Lu DX, Qi RB, Wang HD. Berberine inhibits doxorubicin-triggered cardiomyocyte apoptosis via attenuating mitochondrial dysfunction and increasing Bcl-2 expression. PLoS ONE.

[113] Wang M, Wang J, Tan R, Wu Q, Qiu H, Yang J, Jiang Q (2013). Effect of berberine on PPAR α/NO activation in high glucose- and insulin-induced cardiomyocyte hypertrophy. Evid Based Complement Altern.

[114] Zhang T, Yang S, Du J (2014). Protective effects of berberine on isoproterenol-induced acute myocardial ischemia in rats through regulating HMGB1-TLR4 axis. Evid Based Complement Altern.

[115] Durairajan SSK, Liu LF, Lu JH, Chen LL, Yuan Q, Chung SK, Huang L, Li XS, Huang JD, Li M (2012). Berberine ameliorates *β*-amyloid pathology, gliosis, and cognitive impairment in an Alzheimer’s disease transgenic mouse model. Neurobiol Aging.

[116] Liu YQ, Cheng MC, Wang LX, Xiao HB (2010). Rhizoma Coptidis and berberine-induced activation of murine microglia N9 cells. J Ethnopharmacol.

[117] Zhu X, Guo X, Mao G, Gao Z, Wang H, He Q, Li D (2013). Hepatoprotection of berberine against hydrogen peroxide-induced apoptosis by upregulation of Sirtuin 1. Phytother Res..

[118] Lan T, Liu W, Xie X, Huang K, Peng J, Huang J, Shen X, Liu P, Yang H, Huang H (2012). Berberine suppresses high glucose-induced TGF-beta1 and fibronectin synthesis in mesangial cells through inhibition of sphingosine kinase 1/AP-1 pathway. Eur J Pharmacol.

[119] Tang QL, Lai ML, Zhong YF, Wang AM, Su JK, Zhang MQ (2013). Antinociceptive effect of berberine on visceral hypersensitivity in rats. World J Gastroenterol.

[120] Zhang Q, Piao XL, Piao XS, Lu T, Wang D, Kim SW (2011). Preventive effect of *Coptis chinensis* and berberine on intestinal injury in rats challenged with lipopolysaccharides. Food Chem Toxicol.

[121] Feng AW, Yu C, Mao Q, Li N, Li QR, Li JS (2011). Berberine hydrochloride attenuates cyclooxygenase-2 expression in rat small intestinal mucosa during acute endotoxemia. Fitoterapia.

[122] Tian P, Ge HY, Liu HT, Kern TS, Du LL, Guan LN, Su S, Liu P (2013). Leukocytes from diabetic patients kill retinal endothelial cells: effects of berberine. Mol Vision..

[123] Zhang S, Zhang B, Dai W, Zhang X (2011). Oxidative damage and antioxidant responses in *Microcystis aeruginosa* exposed to the allelochemical berberine isolated from golden thread. J Plant Physiol.

[124] Morita Y, Nakashima K, Nishino K, Kotani K, Tomida J, Inoue M, Kawamura Y (2016). Berberine is a novel type efflux inhibitor which attenuates the MexXY-mediated aminoglycoside resistance in *Pseudomonas aeruginosa*. Front Microbiol..

[125] Ning N, He K, Wang Y, Zou Z, Wu H, Li X, Ye X (2015). Hypolipidemic effect and mechanism of palmatine from *Coptis chinensis* in hamsters fed high-fat diet. Phytother Res..

[126] Lee WC, Kim JK, Kang JW, Oh WY, Jung JY, Kim YS, Jung HA, Choi JS, Lee SM (2010). Palmatine attenuates D-galactosamine/lipopolysaccharide-induced fulminant hepatic failure in mice. Food Chem Toxicol.

[127] Lee JW, Mase N, Yonezawa T, Seo HG (2010). Palmatine attenuates osteoclast differentiation and function through inhibition of receptor activator of nuclear factor-kB ligand expression in Osteoblast Cells. Biol Pharm Bull.

[128] Vrba J, Havlikova M, Gerhardova D, Ulrichova J (2014). Palmatine activates AhR and upregulates CYP1A activity in HepG2 cells but not in human hepatocytes. Toxicol In Vitr.

[129] Kim YM, Ha YM, Jin YC, Shi LY, Lee YS, Kim HJ, Seo HG, Choi JS, Kim YS, Kang SS, Lee JH, Chang KC (2009). Palmatine from Coptidis rhizoma reduces ischemia-reperfusion-mediated acute myocardial injury in the rat. Food Chem Toxicol.

[130] Zhao H, Zhou S, Zhang M, Feng J, Wang S, Wang D, Geng Y, Wang X (2016). An in vitro AChE inhibition assay combined with UF-HPLC–ESI-Q-TOF/MS approach for screening and characterizing of AChE inhibitors from roots of *Coptis chinensis* Franch. J Pharm Biomed..

[131] Jia F, Zou G, Fan J, Yuan Z (2010). Identification of palmatine as an inhibitor of West Nile virus. Arch Virol..

[132] Wu J, Zhang H, Hu B, Yang L, Wang P, Wang F, Meng X (2016). Coptisine from *Coptis chinensis* inhibits production of inflammatory mediators in lipopolysaccharide-stimulated RAW 264.7 murine macrophage cells. Eur J Pharmacol.

[133] Zou ZY, Hu YR, Ma H, Wang YZ, He K, Xia S, Wu H, Xue DF, Li XG, Ye XL (2015). Coptisine attenuates obesity-related inflammation through LPS/TLR-4-mediated signaling pathway in Syrian golden hamsters. Fitoterapia.

[134] Friedemann T, Schumacher U, Tao Y, Leung AK, Schroder S (2015). Neuroprotective activity of coptisine from *Coptis chinensis* (Franch). Evid Based Complement Altern.

[135] Yue Y, Dou L, Wang X, Xue H, Song Y, Li X (2015). Screening β_1_AR inhibitors by cell membrane chromatography and offline UPLC/MS method for protecting myocardial ischemia. J Pharm Biomed..

[136] Liu RF, Cao ZF, Pan YY, Zhang GC, Yang P, Guo PD, Zhou QS (2013). Jatrorrhizine hydrochloride inhibits the proliferation and neovascularization of C8161 metastatic melanoma cells. Anti Cancer Drug.

[137] Yang WW, She LP, Yu K, Yan S, Zhang XF, Tian XY, Ma SR, Zhang XW (2016). Jatrorrhizine hydrochloride attenuates hyperlipidemia in a high-fat diet-induced obesity mouse model. Mol Med Rep..

[138] Zhang B, Cao A, Zhou J, Hu Z, Wu D (2012). Effect of jatrorrhizine on delayed gastrointestinal transit in rat postoperative ileus. J Pharm Pharmacol.

[139] Luo T, Shen XY, Li S, Ouyang T (2016). The protective effect of jatrorrhizine against oxidative stress in primary rat cortical neurons. CNS Neurol Disord DR.

[140] Choi JS, Kim JH, Ali MY, Jung HJ, Min BS, Choi RJ, Kim GD, Jung HA (2015). Anti-adipogenic effect of epiberberine is mediated by regulation of the Raf/MEK1/2/ERK1/2 and AMPKalpha/Akt pathways. Arch Pharm Res..

[141] Bao M, Cao Z, Yu D, Fu S, Zhang G, Yang P, Pan Y, Yang B, Han H, Zhou Q (2012). Columbamine suppresses the proliferation and neovascularization of metastatic osteosarcoma U2OS cells with low cytotoxicity. Toxicol Lett.

[142] Cui L, Liu M, Chang X, Sun K (2016). The inhibiting effect of the Coptis chinensis polysaccharide on the type II diabetic mice. Biomed Pharmacother.

[143] Yang Y, Li Y, Yin DK, Chen S, Gao XD (2016). *Coptis chinensis* polysaccharides inhibit advanced glycation end product formation. J Med Food.

[144] Jiang S, Du P, An L, Yuan G, Sun Z (2013). Anti-diabetic effect of *Coptis Chinensis* polysaccharide in high-fat diet with STZ-induced diabetic mice. Int J Biol Macromol.

[145] Jiang S, Wang Y, Ren D, Li J, Yuan G, An L, Du P, Ma J (2015). Antidiabetic mechanism of *Coptis chinensis* polysaccharide through its antioxidant property involving the JNK pathway. Pharm Biol..

[146] Yan H, Sun X, Sun S, Wang S, Zhang J, Wang R, An P, Yang F, Kang W (2011). Anti-ultraviolet radiation effects of *Coptis chinensis* and *Phellodendron amurense* glycans by immunomodulating and inhibiting oxidative injury. Int J Biol Macromol.

[147] Ma BL, Ma YM, Shi R, Wang TM, Zhang N, Wang CH, Yang Y (2010). Identification of the toxic constituents in rhizoma Coptidis. J Ethnopharmacol.

[148] Yu S, Yu Y, Liu L, Wang X, Lu S, Liang Y, Liu X, Xie L, Wang G (2010). Increased plasma exposures of five protoberberine alkaloids from Coptidis rhizoma in streptozotocin-induced diabetic rats: is P-GP involved?. Planta Med.

[149] Ma BL, Yao MK, Zhong J, Ma YM, Gao CL, Wu JS, Qiu FR, Wang CH, Wang XH (2012). Increased systemic exposure to rhizoma coptidis alkaloids in lipopolysaccharide-pretreated rats attributable to enhanced intestinal absorption. Drug Metab Dispos.

[150] Ma BL, Yin C, Zhang BK, Dai Y, Jia YQ, Yang Y, Li Q, Shi R, Wang TM, Wu JS, Li YY, Lin G, Ma YM (2016). Naturally occurring proteinaceous nanoparticles in Coptidis rhizoma extract act as concentration-dependent carriers that facilitate berberine absorption. Sci Rep..

[151] Linn YC, Lu J, Lim LC, Sun H, Sun J, Zhou Y, Ng HS (2012). Berberine-induced haemolysis revisited: safety of rhizoma Coptidis and Cortex phellodendri in chronic haematological diseases. Phytother Res..

[152] Yi J, Ye X, Wang D, He K, Yang Y, Liu X, Li X (2013). Safety evaluation of main alkaloids from rhizoma Coptidis. J Ethnopharmacol.

[153] Zhou YT, Liao QF, Lin MN, Deng XJ, Zhang PT, Yao MC, Zhang L, Xie ZY (2014). Combination of ^1^H NMR- and GC–MS-based metabonomics to study on the toxicity of Coptidis Rhizome in rats. PLoS ONE.

[154] Ning N, Wang YZ, Zou ZY, Zhang DZ, de Wang Z, Li XG (2015). Pharmacological and safety evaluation of fibrous root of rhizoma Coptidis. Environ Toxicol Pharmacol.

[155] Schramm A, Baburin I, Hering S, Hamburger M (2011). HERG channel inhibitors in extracts of Coptidis rhizoma. Planta Med.

